# Permeability and driving force: why is it difficult to control glycolytic flux by blocking lactate transporters?

**DOI:** 10.18632/oncotarget.28351

**Published:** 2023-01-26

**Authors:** Wiktoria Blaszczak, Pawel Swietach

**Keywords:** MCT inhibitors, fermentative metabolism, lactate, PDAC, SLC16A1

The addiction of cancer cells to glycolysis, even under sufficient oxygenation, is a hallmark of cancer metabolism [1]. Although less energetically efficient than oxidative phosphorylation, glycolysis can service the growth needs of neoplasms, especially in rapidly expanding tumours, where dysfunctional perfusion creates hypoxic niches that restrict mitochondrial respiration. A plausible reason for adopting a glycolytic phenotype is that it allows cells to become more independent of oxygen tension, a labile environmental variable that limits the range of habitats for other types of cells. Aside from providing a steady (albeit reduced) flow of energy, glycolysis facilitates biomass synthesis through its connection with the pentose phosphate pathway. In the absence of oxidative phosphorylation, glycolytic cells produce lactic acid, which is released into the extracellular space as lactate and H^+^ ions aboard H^+^-monocarboxylate transporters (MCTs), alongside a smaller flux of un-ionised lactic acid across the lipid matrix [2]. This creates profound lactic acidosis in the tumour microenvironment which, in turn, contributes towards selection of more invasive phenotypes and evasion of immune surveillance [3]. Upregulated glycolysis has been linked to chemotherapy and immunotherapy resistance [4], and rescues cancer cells from pro-apoptotic signalling [5]. Overall, glycolysis facilitates tumour proliferation and survival, and has become a hotly-pursued target for therapeutic inhibition.

In our recent study (Blaszczak et al. (2022)), using a panel of pancreatic ductal adenocarcinoma cell lines, we characterised how extracellular acidity feeds back to inhibit further glycolytic acid production [6]. In closed compartments, or spaces with limited exchange (like poorly perfused tumours), this feedback circuit results in the attainment of a low extracellular pH at steady-state. The sensor operating this mechanism resides in the cytoplasm and is triggered in acidic environments through the coupling between intra- and extracellular pH. Influencing these sensors pharmacologically is an attractive strategy to control glycolytic metabolism, but accessibility is problematic. To that end, blocking glycolysis by MCT inhibition has been postulated as a realistic alternative. However, we found that MCT inhibitors, regardless of isoform selectivity, have only limited capacity to reduce glycolytic flux at steady-state. This counter-intuitive observation can be reconciled if we consider the factors determining flux: permeability and driving force. MCT inhibitors work by reducing the membrane’s permeability to lactate and H^+^ ions (effectively, lactic acid). A sudden reduction in MCT-dependent permeability will initially reduce glycolytic flux, but in a cell that continually generates lactic acid, a change in driving force is inevitable. Critically, driving force relates to the difference in concentrations on either side of the membrane. For example, in a system with infinitely large permeability, intra- and extracellular concentrations will be equal. MCTs are expressed, and often induced by hypoxia (e.g., MCT4), to produce very high permeability. As a result, the concentration difference across the membrane is normally very small, but has capacity to increase substantially; for example, to off-set a decrease in permeability. Thus, following an acute reduction in MCT-dependent permeability, lactate will accumulate in the cytoplasm. In principle, H^+^ ions should also accumulate yet these are tightly regulated by pH regulators that effectively clamp intracellular pH. Overall, a decrease in permeability will increase driving force, thereby restoring flux. The advantage of this phenomenon, called auto-regulation, is that it stabilizes flux, irrespective of perturbations to transporter permeability, i.e. a potential vulnerability. From a therapy viewpoint, an unwanted consequence of auto-regulation is that inhibitors of permeability (i.e., MCT blockers) cannot produce a proportional decrease in flux, and much larger concentrations of inhibitor may be required, potentially outside realistic dosage regimes. Our findings thus illustrate the importance of an integrative understanding of biological systems.

The problem of flux auto-regulation shifts attention from inhibiting lactate transporters to targeting glycolysis, particularly its rate-limiting enzymes. It would be intriguing to seek ways of selectively acidifying cancer cells, without acidifying their microenvironment. Regardless of the chosen approach, it is likely that any successful therapeutic strategy for targeting glycolysis will be multifaceted to overcome some of the intricacies of complex pathways.
